# Accelerated Oxidative Degradation of Polystyrene: Correlating UV Aging with Reactive Molecular Dynamics

**DOI:** 10.3390/molecules31101730

**Published:** 2026-05-19

**Authors:** Sylwia Pasieczna-Patkowska, Marcin Cichy, Monika Panczyk, Krzysztof Nieszporek, Tomasz Panczyk

**Affiliations:** 1 Department of Chemical Technology, Institute of Chemical Sciences, Faculty of Chemistry, Maria Curie-Sklodowska University in Lublin, pl. Maria Curie-Sklodowska 3, 20031 Lublin, Poland; sylwia.pasieczna-patkowska@mail.umcs.pl (S.P.-P.); marcin.cichy@umcs.pl (M.C.); monika.panczyk@umcs.pl (M.P.); 2 Department of Theoretical Chemistry, Institute of Chemical Sciences, Faculty of Chemistry, Maria Curie-Sklodowska University in Lublin, pl. Maria Curie-Sklodowska 3, 20031 Lublin, Poland; krzysztof.nieszporek@mail.umcs.pl; 3 Jerzy Haber Institute of Catalysis and Surface Chemistry, Polish Academy of Sciences, ul. Niezapominajek 8, 30239 Cracow, Poland

**Keywords:** polystyrene, EPS, ReaxFF, degradation, FT-IR, surface

## Abstract

This study investigates the oxidative degradation of polystyrene (PS) through a synergistic framework integrating UV-C-accelerated aging with Reactive Molecular Dynamics (ReaxFF) simulations. To bridge the gap between experimental and computational timescales, shock compression was employed in the simulations as an accelerator of degradation reactions. ATR-FTIR spectroscopy revealed the emergence of carbonyl (1717 cm^−1^) and peroxyester (1760 cm^−1^) bands, alongside dominant ether-type oxygen bridges (1260, 1209 cm^−1^). These experimental data, particularly the depletion of native aromatic bands (1492, 1451 cm^−1^), provide direct empirical validation of the ring-ring cross-linking and radical-mediated oxidation pathways predicted by the ReaxFF model. The results demonstrate that theory-guided diagnostics offer a robust mechanism for understanding the atomic-level restructuring of the polymer matrix. Significantly, the formation of hydrophilic oxygenated groups increases the bioavailability and environmental hazard potential of fragmented PS microplastics, providing critical insights into their long-term ecological fate.

## 1. Introduction

Global production of plastics has increased dramatically over the past decades, reaching approximately 400–460 million tonnes annually in recent years. This represents nearly a 200-fold increase compared to production levels in the 1950s. The continuous growth in plastic manufacturing is primarily driven by increasing demand in packaging, construction, automotive, and consumer goods sectors [1,2,3]. Polyethylene and polypropylene constitute the largest share of global production, followed by polyvinyl chloride, polyethylene terephthalate, and polystyrene-based materials [3,4]. Despite the widespread use of plastics, global recycling rates remain relatively low. According to a recent assessment, only about 8% of the plastic waste generated in 2023 was effectively recycled, with the majority either landfilled, mismanaged, or entering the environment, posing significant ecological and health concerns [3,5].

Polystyrene (PS) and its expanded form (EPS) account for approximately 6% to 7% of total global plastic production [6,7]. Despite its versatility and low production cost, the environmental footprint of PS is exacerbated by its exceptionally low recovery rates. While materials like PET show higher recyclability, PS often exhibits a recovery rate of less than 1% [8]. Conventional mechanical recycling of PS is frequently hindered by the presence of hazardous chemical additives, such as flame retardants and UV stabilizers, which make the resulting recyclates more recalcitrant to further processing and biodegradation [9,10]. Furthermore, the bulky nature and low density of EPS waste make long-distance transportation economically unfeasible, often leading to its direct disposal in landfills [11]. While chemical recycling through catalytic pyrolysis offers a potential pathway for monomer recovery, these methods typically require prohibitive temperatures (exceeding 300 °C) and complex reactor designs, resulting in high operational costs [12]. Consequently, the vast majority of PS waste persists in the environment, where it undergoes fragmentation into persistent micro- and nanoplastics that pose significant ecological and health risks [13].

Computational modeling has become an indispensable tool for elucidating the mechanisms of polymer degradation at the molecular level, complementing experimental studies and providing detailed insight into reaction pathways, energetics, and kinetics that are difficult to access empirically. Density Functional Theory (DFT) has been widely applied to characterize reaction intermediates, calculate activation energies, and explore bond scission mechanisms in polymer degradation, particularly for processes such as catalytic pyrolysis and oxidative decomposition of polystyrene and other plastics, thereby offering quantitative predictions of reaction energetics and possible pathways for sustainable recycling strategies [14,15,16,17]. The drawback of quantum-chemical methods, however, is the limitation on the system size and the number of atoms that can be practically handled.

Reactive molecular dynamics (MD) simulations using force fields such as Tersoff [18], AIREBO [19,20] or ReaxFF [21] have been successfully utilized to model the dynamic evolution of polymer structures under external stress or shock conditions, capturing bond breakage, radical formation, and crosslinking events in polymers such as polypropylene, polystyrene and others, thus elucidating the molecular-level degradation mechanisms and complementing static quantum calculations [22,23,24,25].

The ReaxFF force field is based on the bond-order formalism, which establishes a relationship between bond order and bond energy, enabling accurate and continuous simulation of bond formation and dissociation processes. Within this framework, all connectivity-dependent interactions, including bond stretching, angle bending, and torsional terms, are formulated as bond-order-dependent functions. Consequently, the contributions of these terms gradually decrease as bonds weaken or break, allowing for a realistic description of reactive processes. In contrast, nonbonded interactions, such as van der Waals and Coulomb forces, are calculated between all pairs of atoms regardless of their connectivity. Although these interactions do not explicitly depend on bond order, they strongly depend on interatomic distances and therefore must be updated at every simulation step. Furthermore, ReaxFF determines atomic charges using a geometry-dependent charge equilibration scheme based on the electronegativity equalization method, enabling dynamic redistribution of charge during chemical reactions [25,26].

The ReaxFF formalism does not rely on predefined atom types but instead employs a single parameter set to describe each element, independent of its local chemical environment. As a result, ReaxFF parameterization is strongly dependent on the chemical context of the studied reactions, leading to the development of multiple parameter sets tailored to specific chemical systems. In particular, ReaxFF parameters optimized for high-temperature and high-pressure conditions are generally not transferable to aqueous environments or, more broadly, to mild physical conditions [25,26].

The natural degradation of plastics is a slow process occurring under relatively mild physical conditions, particularly with respect to temperature and pressure. Therefore, laboratory experiments on plastic degradation are often conducted under more severe conditions. In particular, natural sunlight is frequently replaced with artificial UV radiation to accelerate processes that would otherwise take months or even years [27,28]. High-energy UV radiation activates chemical bonds and generates free radicals, effectively creating extreme physicochemical conditions for the sample. Consequently, molecular modeling studies, including those employing ReaxFF, also require acceleration techniques to enable observation of processes that would otherwise exceed the accessible simulation timescales.

Shock-compressed material is characterized by an extremely rapid and intense increase in pressure, temperature, and density. This increase propagates through the material at supersonic velocity. The thermodynamic properties associated with shock waves are commonly described using the Rankine–Hugoniot jump conditions, which are derived from the conservation of mass, momentum, and energy [29,30]. Owing to their simplicity and effectiveness, these relations are widely used to quantitatively determine key shock parameters, including pressure, density, and internal energy both ahead of and behind the shock front, as well as the shock-wave and particle velocities. Consequently, shock compression represents a highly effective approach for accelerating possible chemical transformations within the material.

Expanded polystyrene (EPS) is widely used in both construction and packaging applications due to its excellent insulation properties and low cost, but these same characteristics contribute to its persistence as a problematic waste stream [12,31]. EPS is non-biodegradable and highly resistant to natural degradation, which leads to its accumulation in landfills and the environment, posing significant challenges for waste management and pollution control [32,33]. Under environmental conditions, EPS undergoes photo-oxidative weathering and physical fragmentation, resulting in the generation of micro- and nanoplastic particles that are readily transported by wind and water and can persist for long periods [7,27,33]. These fragments are of particular concern because they are easily ingested by wildlife and can adsorb and release hazardous chemicals, thereby entering food chains and posing ecotoxicological and human health risks [27]. Furthermore, EPS debris has been shown to induce genotoxic effects in littoral organisms, reinforcing the potential hazard that degraded polystyrene plastics present to both ecosystems and human health [34]. It is therefore important to achieve a comprehensive understanding of the surface chemistry of degraded EPS debris in order to develop effective prevention and mitigation strategies.

Elucidating the exact molecular pathways of PS degradation remains a complex task due to the simultaneous occurrence of various competing reactions [35]. While experimental techniques such as Attenuated Total Reflection Fourier Transform Infrared (FT-IR/ATR) spectroscopy are invaluable for identifying emerging functional groups like carbonyls (1717 cm^−1^), hydroxyls (3386 cm^−1^), and ether bridges (1260 cm^−1^), they often provide only a static or averaged view of the process [36]. Traditional quantum-mechanical (DFT) calculations offer high precision but are computationally prohibitive for modeling the long-term structural restructuring of large polymer matrices.

The photo-oxidative degradation of polystyrene (PS) is widely recognized to proceed via a classical free-radical auto-oxidization chain mechanism, initiated by the absorption of UV radiation by the polymer’s internal chromophores, primarily the pendant phenyl rings [6,31,36]. This photoexcitation leads to the formation of excited singlet and triplet states, which provide sufficient energy to facilitate the homolytic cleavage of C–H bonds. Due to significantly lower bond dissociation energy, the tertiary hydrogen atoms on the polymer backbone are the most susceptible to this abstraction, resulting in the formation of reactive polymer alkyl radicals (P•) [4,31,37]. In the subsequent propagation step, these radicals react rapidly with molecular oxygen (O_2_) to form polymer peroxy radicals (POO•), which continue the chain by abstracting hydrogen from adjacent segments to yield polymer hydroperoxides (POOH). While pure PS lacks chromophores for long-wave UV radiation (λ > 300 nm), initiation in real-world conditions is often triggered by structural defects and internal impurities [4,7,31,35,37]. Specifically, in-chain peroxide linkages (-O-O-) act as “weak links” due to their low dissociation energy, while acetophenone end-groups, formed during polymerization or initial processing, serve as potent initiators by producing reactive radicals through triplet state excitation. The accumulation and subsequent photolysis of these hydroperoxides lead to β-scission of the backbone, causing chain fragmentation, brittle failure, and the characteristic yellowing associated with the formation of oxygenated photoproducts like ketones and aldehydes [11,31].

The aim of this work is to provide a combined experimental and theoretical analysis of the degradation chemistry of polystyrene (PS) in the presence of molecular oxygen. A key outcome of this study is the demonstrated qualitative agreement between both approaches, showing that they consistently describe the same underlying chemical processes and strongly support one another. Both methods employ acceleration techniques: UV-C radiation is used for the experimentally analyzed samples, while shock compression based on Hugoniot dynamics is applied in molecular simulations performed with the ReaxFF force field using the CHON-2019 parameterization [26]. Although these approaches differ in their physical nature, they lead to chemically consistent results, indicating that they probe comparable regions of the degradation pathway. The experimentally degraded samples were analyzed using FT-IR spectroscopy, enabling the identification of new chemical bonds formed during exposure to UV-C radiation or natural sunlight over various time intervals. These experimental observations were directly used to validate the results obtained from molecular simulations. Such validation is particularly important, as the ReaxFF force field often requires careful assessment when describing newly formed chemical species.

The strong qualitative consistency between experimental data and theoretical predictions confirms the reliability of the CHON-2019 parameterization [26] for this system. Moreover, it demonstrates that the proposed combined methodology is a robust tool for investigating polymer degradation, providing detailed insight into the molecular structure of PS degradation in the presence of molecular oxygen.

## 2. Results

### 2.1. FT-IR Analysis of EPS Samples

**Figure 1:**
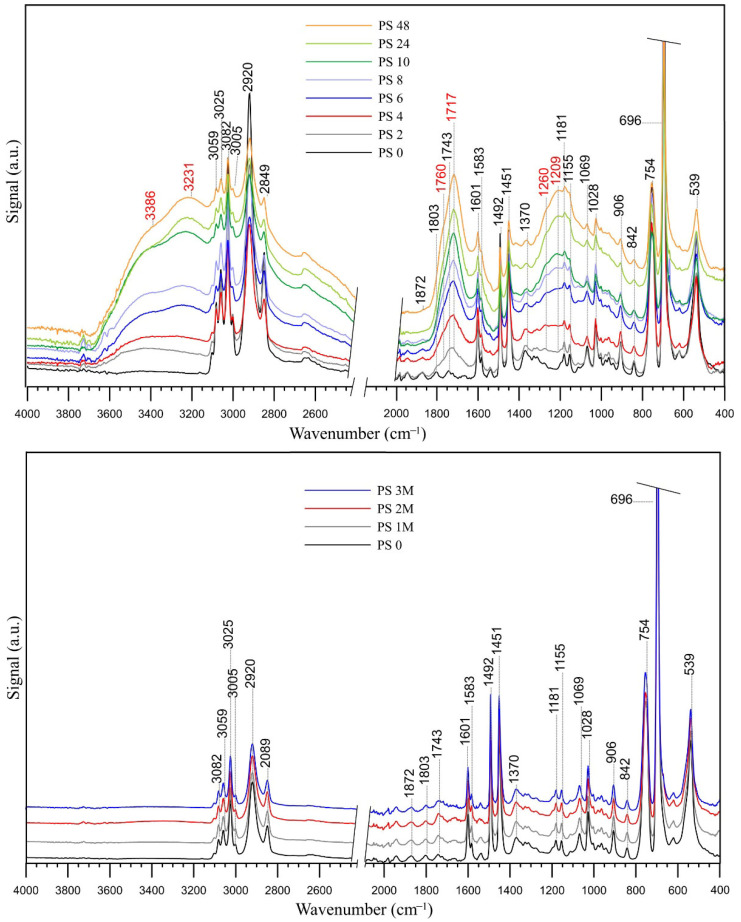
(**Top**) FT-IR spectra of polystyrene (PS) samples subjected to photo-oxidative degradation for various time intervals (0, 2, 4, 6, 8, 10, 24, and 48 h). (**Bottom**) FT-IR spectra of EPS samples exposed to natural sunlight for 3 months. The baselines are displaced vertically to avoid overlap.

The FT-IR spectra of the polystyrene samples in their pristine state and after various intervals of UV irradiation are displayed in Figure 1. The spectral range from 2400 to 2100 cm^−1^ was excluded from the figure as it contains no significant diagnostic information, and its removal enhances the overall clarity and readability of the presented spectra.

The FT-IR spectrum of the pristine polystyrene (PS 0) reveals several fundamental vibrational modes characteristic of its molecular structure. The high-wavenumber region is dominated by C–H stretching vibrations. Specifically, the peaks at 3082, 3059, and 3025 cm^−1^ are attributed to the aromatic =C–H symmetric stretching vibrations of the benzene ring [9,12,31].

The bands observed at 2920 cm^−1^ and 2849 cm^−1^ correspond to the asymmetric and symmetric stretching vibrations of the aliphatic CH_2_ methylene groups in the polymer backbone. In the mid-infrared region, the series of low-intensity peaks between 2000 and 1650 cm^−1^ (specifically 1981, 1943, 1872, and 1803 cm^−1^) represent the characteristic aromatic ring overtones, often referred to as “benzene fingers”. These signals are indicative of a mono-substituted benzene ring [38]. The aromatic C=C stretching vibrations are clearly identified by the notable absorption peaks at 1601 cm^−1^ and 1583 cm^−1^. Furthermore, the intense bands at 1492 cm^−1^ and 1451 cm^−1^ are associated with the stretching vibrations of the aromatic ring coupled with the scissoring vibrations of the methylene groups. The fingerprint region contains several diagnostic bands for the polystyrene skeletal structure. The peaks at 1069 cm^−1^ and 1028 cm^−1^ are related to aromatic C–H in-plane bending and deformation. Most significantly, the very sharp and intense peaks at 754 cm^−1^ and 696 cm^−1^ are attributed to the out-of-plane bending of aromatic C–H bonds and the angular deformation of the C–H groups within the aromatic ring. The presence of these two specific peaks serves as a primary indicator for the mono-substitution of the benzene ring. Additional low-frequency vibrations at 621 cm^−1^ and 539 cm^−1^ correspond to ring out-of-plane deformation [31,38].

As illustrated in the bottom part of Figure 1, exposure of the samples to natural sunlight for up to three months did not lead to substantial changes in the FT-IR spectra when compared to the PS 0 sample. This indicates that photodegradation of PS is a relatively slow process within the typical timeframe of laboratory experiments. Therefore, accelerated degradation processes induced by UV-C irradiation are required to observe any chemical modifications of PS. The results of such experiments for various irradiation times are presented in the upper part of Figure 1.

The photo-oxidation of PS leads to significant structural modifications, primarily driven by free radical chain reactions that introduce oxygenated functional groups into the polymer matrix [11,31]. The FT-IR spectra of the samples subjected to degradation for up to 48 h exhibit the emergence of several diagnostic bands at 3386, 3231, 1760, 1717, 1260, and 1209 cm^−1^, which confirm the progression of oxidative chain scission and the formation of secondary photoproducts. The broad absorption features appearing in the high-wavenumber region indicate the formation of hydroxyl-containing species. The band at 3386 cm^−1^ is attributed to the O–H stretching vibrations of associated alcohols or hydroperoxide intermediates (–OOH) [35,39]. These species are primary products of the reaction between polymer radicals and ambient oxygen [12,36,39]. The peak at 3231 cm^−1^ is characteristic of dimerized carboxylic acids (–COOH dimers). The presence of these dimers suggests advanced oxidation and the formation of acidic end-groups following the cleavage of the polymer backbone [35,39].

The most prominent evidence of degradation is found in the carbonyl stretching region, where two distinct peaks emerge. The sharp and intense peak at 1717 cm^−1^ corresponds to the C=O stretching of aliphatic ketones or carboxylic acids. Literature identifies this specific wavenumber (often reported between 1716 and 1720 cm^−1^) as a major indicator of oxygen incorporation into the polystyrene matrix during UV exposure [9,28,31,36]. Aligned with the integrated computational-experimental framework of this study, the band at 1760 cm^−1^ is assigned to peroxyesters. While this region is frequently linked to γ-lactones in traditional PS aging studies, our results (further elucidated in Section 2.2) and recent literature [28] strongly point toward organic peroxy species.

The formation of oxygenated species is further corroborated by changes in the fingerprint region. The peak at 1260 cm^−1^ is attributed to the asymmetric C–O stretching in esters or carboxyl groups. Similar bands in this region have been associated with the formation of low-molecular-weight phthalates and other fragmentation products during photocatalytic or UV-induced degradation [12,40]. The peak at 1209 cm^−1^ corresponds to the C–O stretching vibrations or skeletal vibrations of carbon atoms adjacent to a carbonyl group.

The experimental data reveals that the intensity of these oxygen-related bands increases progressively with irradiation time, particularly after 10 h of exposure. This rise is accompanied by a concurrent decrease in the intensity of native PS bands, such as the aromatic ring vibrations at 1492 and 1451 cm^−1^, signaling the efficient breakdown of the polymer backbone and the depletion of the original structure. These findings are consistent with a Norrish-type degradation mechanism and hydroperoxide photolysis, leading to the accumulation of ketones, aldehydes and lactones [28,36].

### 2.2. Molecular Dynamics Simulation of PS Degradation in the Presence of Oxygen

#### 2.2.1. Generation of Intact Polystyrene Nanoparticles for MD Simulations of Degradation

**Figure 2:**
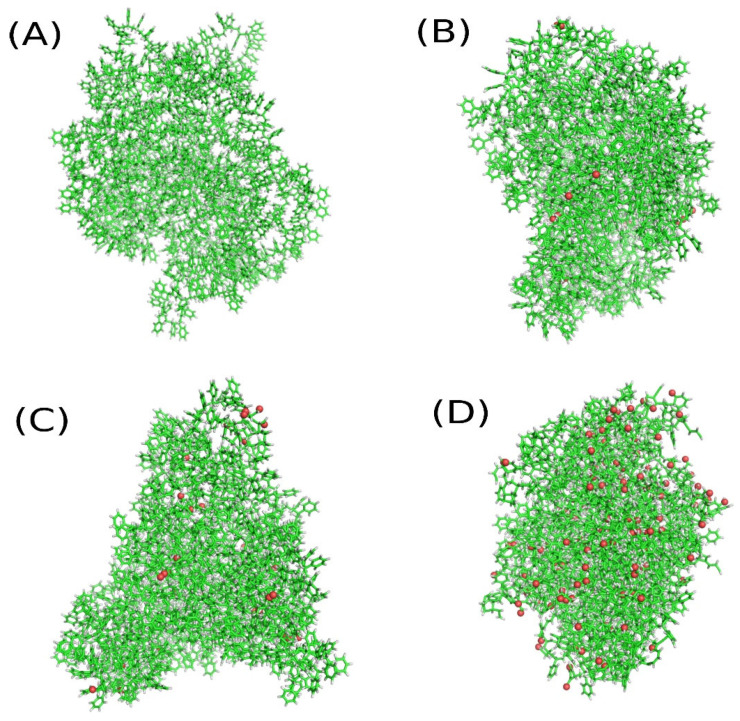
(**A**) The initial structure of the polystyrene (PS) nanoparticle obtained using the AMBER force field, as well as its structure after shock compression at 50 GPa. (**B**) The structure of the degraded PS nanoparticle after shock compression at 60 GPa, while panels (**C**,**D**) correspond to compression pressures of 70 and 80 GPa, respectively. Carbon atoms are shown as green sticks, hydrogen atoms as gray sticks, and oxygen atoms bonded to the PS nanoparticle as a result of reactions with molecular oxygen are shown as red spheres.

A single polystyrene (PS) chain was generated using the *tleap* program from the AmberTools23 package [41]. The polymer was built from a monomer unit for which atomic point charges and AMBER force field parameters were generated using the RED Server Development [42,43]. The polymer chain consisted of 193 monomer units and had a molar mass of 20,130 g·mol^−1^, corresponding to the molecular formula C_1546_H_1550_. Three such polymer chains were placed in the simulation box with approximately mutually perpendicular orientations and subjected to a standard molecular dynamics simulation in the NVT ensemble, allowing the initially linear chains to fold into a compact conformation. The resulting structure constituted a polystyrene nanoparticle and was used as the initial configuration for subsequent degradation simulations. This structure is shown in Figure 2. This stage of the simulation was used solely to obtain the atomic coordinates of the PS atoms, and any information related to the AMBER force field was discarded in subsequent calculations focusing on PS degradation.

#### 2.2.2. Degradation of the Polystyrene Nanoparticle in the Presence of Oxygen

In dry conditions, polystyrene (PS) is typically exposed to air, and its degradation is therefore expected to involve oxidation reactions with atmospheric oxygen. These processes can be further accelerated by UV radiation, which may lead to the formation of reactive oxygen species as well as radical sites on the surface atoms of the PS material. As already mentioned, chemical reactions cannot be accurately modeled using classical force fields such as AMBER, which do not allow for bond breaking or formation. The most appropriate tools for describing chemical reactivity are quantum chemical methods; however, despite their high accuracy, they are generally limited to relatively small systems due to their high computational cost.

An alternative approach is provided by reactive molecular dynamics, i.e., molecular dynamics simulations employing reactive force fields, which offer a suitable theoretical framework for modeling chemical reactions in large-scale systems. This approach comes at the cost of a somewhat less detailed description of individual reaction events and the lack of explicit treatment of electronic structure and electron transfer processes.

Thus, the simulation box was constructed by placing an intact PS nanoparticle and introducing one hundred oxygen molecules. The force field of the mixed system was then switched to ReaxFF with the CHON-2019 parameterization [26], and the simulation was run for 4 ns under constant-volume (NVT) conditions at 300 K and 1 bar pressure in order to equilibrate both the PS structure and the oxygen molecules within the new force-field framework. Despite the high reactivity of molecular oxygen, no reactions between oxygen and the PS nanoparticle were observed during this equilibration stage. This is expected, as spontaneous oxidation reactions proceed very slowly at ambient temperatures in the absence of external activation.

It should be noted that the current model does not account for the presence of various additives in the polymer structure, such as stabilizers, pigments, or other impurities. Furthermore, the surrounding environment of the PS nanoparticle is limited to molecular oxygen only. Including additional components, such as water, nitrogen, or other constituents of air, would significantly increase the elemental complexity of the ReaxFF force field and require dedicated parameterization. This, in turn, would greatly complicate both the simulations and the interpretation of the results. Therefore, in this study, we deliberately focus on the simplest scenario, which allows us to track the most important descriptors of the degradation process rather than attempting to reproduce the full complexity of real-world conditions.

To initiate reactions, the system was subjected to shock compression at various pressures. This stage of the calculation was performed using LAMMPS [44] with a specialized barostat, *fix nphug*, designed to satisfy the Hugoniot equation of state [29]. Compression pressures were gradually increased, and the molecular composition of the simulation box was monitored. The lowest pressure at which the number of oxygen molecules began to decrease was identified as the threshold pressure for initiating chemical reactions in the system. This pressure was 50 GPa; therefore, the production simulations started from a compression pressure of 50 GPa and included three slightly higher pressures, namely 60, 70, and 80 GPa. The highest pressure, 80 GPa, was chosen based on the requirement that all oxygen molecules be consumed in the reaction occurring at that pressure.

**Figure 3:**
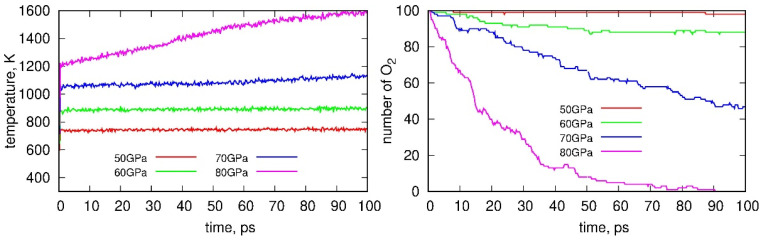
(**Left**) Time evolution of the sample temperature during shock compression at different compression pressures. (**Right**) The number of unreacted oxygen molecules in the simulation box under the same conditions.

As quantitative descriptors of the degradation reactions (reactions with oxygen) of the PS nanoparticle, we selected the system temperature induced by shock compression and the number of remaining oxygen molecules. Both descriptors are shown in Figure 3. At 50 GPa, the compression leads to significant heating of the sample to approximately 750 K, and the temperature then remains constant, indicating that a steady state is established. Similarly, higher compression pressures result in higher temperatures, which again reach steady-state values, at least up to 70 GPa. In contrast, the highest compression pressure exhibits a continuously increasing temperature trend up to 90 ps, after which a plateau is observed. The temperature increase during shock compression results from elevated atomic velocities induced by the passage of the shock wave. This, in turn, facilitates overcoming activation barriers that normally limit chemical reactions under ambient conditions. Consequently, shock compression enables the gradual initiation of reactions characterized by significant activation barriers and inherently slow reaction rates.

The number of oxygen molecules in the simulation box remains almost constant at 50 GPa, indicating that reactions rarely occur at this pressure. As the compression pressure increases, the number of oxygen molecules decreases more rapidly, reflecting an enhanced reaction rate. At the highest compression pressure, all oxygen molecules are consumed as a result of the reactions. However, other phenomena, such as fragmentation of the sample, also occur and will be discussed in detail later.

In the next stage of the degradation simulation, the shock compression was stopped and the samples were allowed to relax and expand under constant-volume and constant-temperature (300 K) conditions. During this stage, reactions within the simulation box were still occurring, but they predominantly proceeded in the reverse direction. This behavior arises because strong compression inhibits the breaking of certain bonds due to external compressive forces. Once the compression is released, these bonds can break more readily, leading to extensive structural reorganization within the bond topology.

Therefore, after completing the relaxation stage, the samples were additionally subjected to annealing at 800 K to remove weak and transient connections that typically form after strong compression when using the ReaxFF force field. The final stage involved cooling the samples to 300 K; by this point, the chemical composition of the systems had stabilized.

Figure 2 qualitatively illustrates how the structure of the PS nanoparticle is altered by compression in the presence of molecular oxygen. Panel (A) shows the state after compression to 50 GPa and the subsequent treatment, which ultimately leads to recovery of a structure identical to that before shock compression, i.e., the initial state of the PS nanoparticle. Panels (B–D) show progressively more altered structures corresponding to increasing compression pressures. In these snapshots, oxygen atoms are shown as red spheres and represent atoms that became chemically bonded to the PS nanoparticle as a result of reactions with molecular oxygen.

#### 2.2.3. Analysis of the Chemical Composition of Degraded PS Nanoparticles

The number of reacted oxygen molecules is one of the key indicators of chemical transformations occurring during nanoparticle degradation simulated by shock compression. However, the subsequent relaxation and annealing stages led to a partial recovery of molecular oxygen content, as some of the newly formed connections corresponded to weakly bound, transient bonds. Therefore, a detailed analysis of the bond topology of the PS nanoparticles is required once the system reaches its final stable chemical state.

**Figure 4:**
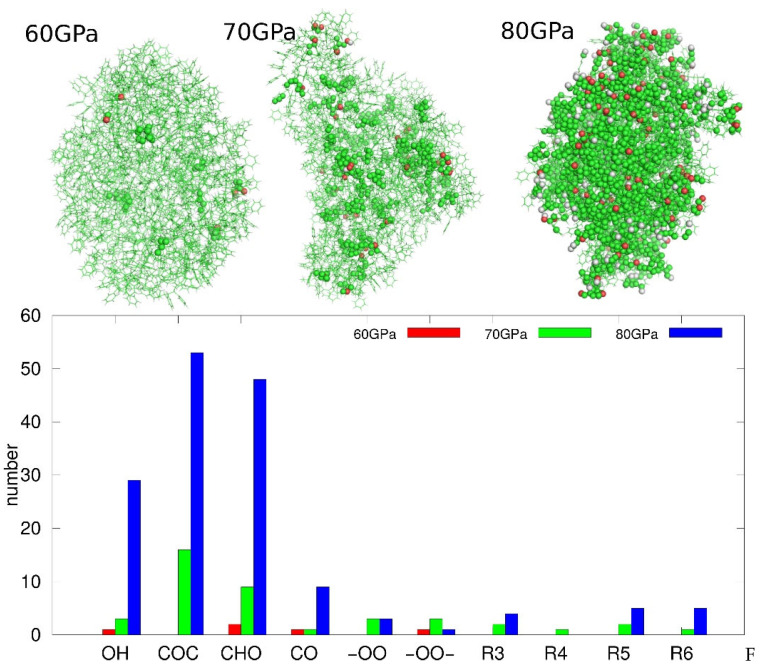
(**Top**) Snapshots highlighting atoms (as spheres) whose chemical environments were altered in any way during degradation at various compression pressures. (**Bottom**) Bar charts showing the populations of the most abundant oxygen-containing functional groups identified in samples obtained at different compression pressures.

Analysis of the PS sample subjected to 50 GPa compression revealed that, after the annealing stage, all oxygen molecules were recovered and the sample reverted to an intact PS state, comparable to that prior to degradation. This indicates that the conditions generated by shock compression up to 50 GPa were insufficient to initiate permanent chemical reactions with oxygen. To facilitate comparison of the chemical states of PS nanoparticles subjected to different compression pressures, Table 1 summarizes the most important descriptors of the chemical state of the samples. Additionally, Figure 4 illustrates the extent of sample degradation at various compression pressures by highlighting atoms whose local environments were altered in any way during degradation. The figure also presents the populations of newly formed chemical bonds and functional groups generated at different compression pressures.

As shown in Figure 4, the number of atoms whose chemical environment was altered due to degradation induced by shock compression increases rapidly with increasing compression pressure. At 60 GPa, only a few dozen atoms exhibit changes in their local chemical surroundings. Increasing the compression pressure results in a significant rise in the number of such atoms, as observed at 70 GPa. Finally, compression to 80 GPa leads to extensive structural alteration of the sample, with the chemical environments of most atoms being modified. The degradation also leads to an increasing degree of fusion of individual polymer chains into larger supermolecules. Such fusion at 60 GPa is limited, as only two of the three chains are linked together, but at higher compression all three chains become linked into a single supermolecule. At the same time, fragmentation of the nanoparticle also occurs, being relatively limited at 70 GPa and very strong at 80 GPa. These two phenomena lead to non-monotonic changes in both the gyration radius (Rg) of the nanoparticle and the solvent-accessible surface area (SASA). It is observed that Rg does not change until 70 GPa, even though several cross-links have already formed. However, it rises significantly at 70 GPa and decreases at 80 GPa. This behavior is related to two factors: the addition of oxygen atoms from the surroundings to the molecular structure of the nanoparticle, and the simultaneous intensification of fragmentation.

Similarly, SASA decreases with increasing degradation due to the slight shrinkage of the nanoparticles resulting from cross-link formation. However, at 70 GPa, there is a strong increase in SASA due to the roughening of the surface. Finally, a significant decrease in SASA occurs at 80 GPa due to intense cross-linking and fragmentation of the material.

**Figure 5:**
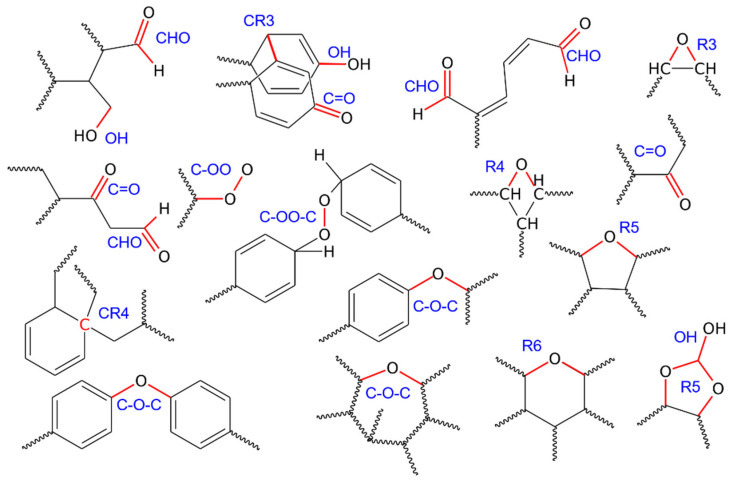
Illustrative representations of the new chemical connections listed in Table 1, identified after degradation of PS. The chemical environments of these groups are taken directly from the bond topology of the PS nanoparticles; however, the depicted environments are not unique, and these groups may occur in other chemical contexts, particularly at higher compression pressures (80 GPa).

Looking at the populations of new chemical groups in Table 1 and Figure 4, we can deduce that the first transformations occurring at 60 GPa correspond to the formation of one hydroxyl group, two aldehyde groups, one carbonyl group, one peroxy linkage, and 39 cross-links of CR3 type. The chemical environments of these new linkages are illustrated in Figure 5.

Thus, degradation is initiated primarily by the formation of CR3 cross-links, which correspond to the development of bonds between neighboring aromatic rings. This type of connection is illustrated in Figure 5 and denoted as CR3. The aromatic rings forming a CR3 cross-link may belong either to the same polymer chain or to different chains; therefore, the decisive factor for cross-link formation is the close spatial proximity of two aromatic rings. It appears that, during PS degradation in a dry environment, aside from the presence of molecular oxygen, the most readily occurring reaction is the formation of CR3 cross-links, as their population is dominant at all investigated compression pressures.

CR4 cross-links are much less frequent but are still present at all compression pressures. They are identified as carbon atoms that form four direct bonds with other carbon atoms, with the additional condition that, prior to degradation, these atoms had only three carbon–carbon bonds. Such carbon atoms are present in the intact PS chain at positions where aromatic rings are connected to the aliphatic backbone of the polymer. Analysis of the bond topology of degraded PS at various compression pressures leads to the conclusion that CR4 atoms appear in multiple chemical contexts and can be formed by linking previously three-bonded carbon atoms to PS chains at randomly distributed locations.

The oxygen-containing groups consist mainly of ether linkages (C–O–C), aldehyde groups (CHO), and hydroxyl groups (OH), as shown in Figure 4. Other types are present in much smaller populations, although their contribution cannot be neglected. C–O–C linkages are the most abundant products of PS degradation under dry conditions in the presence of molecular oxygen. They occur in multiple chemical contexts, three of the most representative examples of which are illustrated in Figure 5. These linkages typically form bridges between different segments of PS chains; in particular, they can connect aromatic rings to each other or link aromatic rings with the aliphatic backbone. C–O–C linkages also appear in more complex topologies, including cyclic arrangements such as the seven-membered ring shown in Figure 5. Rings with six or fewer members were classified as separate groups, although they formally also contain ether linkages. Overall, degradation of PS in the presence of molecular oxygen leads predominantly to the formation of oxygen-mediated links between carbon atoms, namely ether linkages and oxygen-containing rings (R3–R6).

The aldehyde groups are the second most abundant oxygen-containing species formed during PS degradation. Their formation requires chain scission and/or cleavage of aromatic rings, likely due to interactions with oxygen, possibly in the form of radicals. Interestingly, no carboxylic groups were detected alongside the aldehyde functions. Hydroxyl groups, in turn, appear either as a result of chain scission or from the dearomatization of benzene rings, occurring in roughly the same proportions. A similar trend is observed for carbonyl groups, which mainly appear in strongly distorted fragments of the molecule at the highest levels of degradation. An intriguing finding is the presence of peroxy groups, either as linkages within the polymer chain or as terminal groups associated with radicals. These groups are already present at 60 GPa, i.e., at the early stages of degradation, and their population does not increase significantly at higher levels of degradation. They are clearly the result of the “intrusion” of activated oxygen molecules into the polymer network.

An important general conclusion is that PS degradation in the presence of molecular oxygen mainly leads to the formation of oxygen-containing species on the polymer surface rather than combustion, i.e., the production of carbon oxides. Of course, such processes occur at the highest compression, because in addition to the medium- and small-molecule fragments listed in Table 1, many other small molecules are produced, including carbon monoxide, carbon dioxide, water, hydrogen, glyoxal, and several small-molecule radicals. Among these, the most abundant are CO and H_2_, typical products of thermal gasification of organic matter.

## 3. Discussion

Comparison of results from time-dependent FT-IR analysis of UV-C-degraded EPS samples with results obtained from reactive molecular dynamics simulations of shock-compressed PS samples reveals good consistency and mutually complementary insight into the degradation of PS in the presence of oxygen. Table 2 summarizes the most important chemical species identified both in the FT-IR spectra of experimental samples and in the analysis of molecular dynamics simulation results. As indicated by the MD results, the most significant chemical transformation in the PS sample is the formation of cross-links between benzene rings, denoted as CR3 in Table 1, suggesting that structural transformation involves extensive intermolecular ring coupling.

This molecular-scale phenomenon is macroscopically reflected in the FT-IR spectra by a pronounced decrease in the intensity of the native aromatic bands at 1492 cm^−1^ and 1451 cm^−1^ in Figure 1, which correspond to C=C ring vibrations [7,45]. The attenuation of these peaks indicates disruption of the original aromatic symmetry and recombination of phenyl radicals into a more rigid and interconnected polymer network [46,47]. This experimental depletion of the 1492 and 1451 cm^−1^ bands serves as direct empirical verification of the extensive ring-ring (CR3) cross-linking predicted by ReaxFF simulations, confirming that the degradation involves a fundamental atomic-level restructuring of the polymer matrix into a more rigid, interconnected network. However, the large number of CR3 connections may also be facilitated by the specific conditions of shock compression applied here, which act as a biasing factor. Unfortunately, we cannot precisely disentangle what fraction of the CR3 connections arises from the photo-oxidation mechanism and what fraction results from the high pressure associated with the passage of the shock front through the material.

Theoretical results suggest that ether-type oxygen bridges (C–O–C) are the most frequently formed structural modifications during PS degradation. This prediction is empirically validated by the emergence of new absorption bands at 1260 cm^−1^ and 1209 cm^−1^ in Figure 1. These signals correspond to the C–O stretching vibrations of oxidized aliphatic fragments and skeletal vibrations of carbon atoms adjacent to carbonyl groups. The significant intensity of these bands in the 48 h sample confirms that oxygen incorporation is not limited to terminal groups but involves extensive modification of the polymer framework [12,40].

The reactive molecular dynamics identifies aldehydes (CHO) and carbonyls (C=O) as the next most abundant products. In the experimental spectra, this is represented by the intense growth of a sharp band at 1717 cm^−1^. While often generally assigned to ketones [9,31], 2D-COS analysis and literature confirm that this region encompasses a variety of overlapping species, including aliphatic ketones, benzaldehyde, and carboxylic acids [36,46].

The distinct peak observed at 3231 cm^−1^ is assigned to O-H stretching vibrations in dimerized carboxylic acids (-COOH dimers), providing evidence for the advanced stages of photo-oxidative chain scission. This assignment is in agreement with experimental findings by Mailhot and Gardette [35], who identified a similar feature in the 3250 cm^−1^ region for aged polystyrene. While ReaxFF simulations did not produce carboxylic acid groups, they did identify a high abundance of aldehyde (CHO) precursors. Given that carboxylic acids are known secondary oxidation products of aldehydes, the emergence of the 3231 cm^−1^ band suggests that these intermediate species undergo further chemical transformation over timescales that extend beyond the simulated reactive trajectories. This highlights the complementary nature of our approach: ReaxFF elucidates the primary radical-driven formation of oxygenated precursors, while FT-IR confirms the final chemical endpoints of the polystyrene aging process.

The emergence of a distinct band at 1760 cm^−1^ provides significant empirical evidence for the formation of organic peroxide species, such as peroxyesters or peracids, during the photo-oxidative aging of PS. While this region is frequently associated with γ-lactones (cyclic esters), which result from the stabilization of internal radicals during advanced stages of chain scission [28,39], ReaxFF simulations explicitly predicted the formation of peroxide groups (O-O) and peroxy linkages (C-OO-C) rather than cyclic esters. This theoretical finding is strongly supported by recent literature [28], which assigns related features in the 1774 cm^−1^ region to the C=O stretching of organic peroxide groups formed through the interaction of polymer radicals with atmospheric oxygen. The assignment of the 1760 cm^−1^ band to peroxyesters thus establishes a direct correlation between peroxides predicted by reactive molecular dynamics and the experimental vibrational signatures observed in the early-to-mid stages of PS degradation [35]. Alongside the spectral evolutions, the PS samples exhibited a distinct yellow discoloration, which is widely recognized in the literature as a critical macroscopic manifestation of photo-oxidative aging. This physical change strongly correlates with the progressive growth of the carbonyl absorption at 1717 cm^−1^, signaling the accumulation of oxygenated photoproducts such as aliphatic ketones and aromatic derivatives [11,28,31].

Hydroxyl groups were theoretically predicted as a significant product class. This is confirmed by the broad absorption feature at 3386 cm^−1^ in Figure 1, representing O–H stretching in associated alcohols [35,46]. Crucially, the theoretical identification of peroxide groups (O–O) is validated by the broadness of this 3386 cm^−1^ region, which is also assigned to associated hydroperoxides (PS-OOH), key but transient intermediates in the Norrish-type degradation cycles [36,47].

The thermodynamic correlation between experimental UV-C aging and computational shock compression is based on the unified principle of energy injection to overcome the high activation barriers governing polymer degradation. Under ambient conditions, the degradation of polystyrene (PS) is restricted by high dissociation energies, specifically the ~330 kJ mol^−1^ required for tertiary C-H scission [37]. While the physical modes of energy delivery differ—UV-C radiation utilizes electronic excitation through photon absorption (raising molecules to excited singlet or triplet states), whereas shock compression employs Hugoniostat dynamics to rapidly increase atomic velocities (temperature) and mechanical stress—both methods successfully elevate macromolecular segments to energy states that exceed these dissociation thresholds.

The literature indicates that, at least in the case of PS, thermo-oxidation and photo-oxidation follow similar degradation pathways, differing only in their initiation [4]. From a thermodynamic standpoint, light absorption excites the macromolecule into upper vibrational levels in a single step, while the shock wave facilitates the rapid accumulation of vibronic energy through intermolecular collisions, effectively driving the polymer chains toward the same homolytic scission pathways and reactive radical intermediates. In reactive molecular dynamics, these highly accelerated conditions (pressures of 50–80 GPa and temperatures exceeding 1000 K) act as a necessary numerical accelerator to explore the same Potential Energy Surface (PES) sampled during long-term UV exposure, bridging the gap between experimental days and picosecond simulation trajectories. This synergy is empirically validated by the qualitative agreement between predicted pathways and observed chemical fingerprints in 48 h experimental FTIR data. The ReaxFF model identifies primary radical-mediated modifications, such as the formation of ether-type oxygen bridges (validated by bands at 1260 and 1209 cm^−1^) and peroxide intermediates (3386 cm^−1^), which are thermodynamically consistent with the final chemical endpoints. Furthermore, the experimental depletion of native aromatic skeletal bands (1492 and 1451 cm^−1^) signals the disruption of original aromatic symmetry, providing direct empirical proof of the ring-ring (CR3) cross-linking events identified as dominant in the simulations.

Crucially, the formation of small molecules in the simulation trajectories is chemically consistent with standard volatile photoproducts identified in the literature as occurring during low-temperature PS photo-oxidation, rather than being mere artifacts of thermal decomposition or gasification. Specifically, molecular hydrogen is recognized as the primary gaseous product of PS photolysis resulting from homolytic C-H scission and subsequent recombination of highly mobile hydrogen radicals or polyene formation [37,48]. Water is documented as the major volatile photoproduct of styrenic polymers, often produced in quantities exceeding the initial moles of oxygen through hydrogen abstraction by hydroxyl radicals [4,37]. Similarly, carbon monoxide is a well-documented product of Norrish Type I reactions and the secondary scission of hydroperoxides and peroxyesters during photodegradation [37]. This integrated approach proves that although the trigger is different, the resulting atomic restructuring follows the same thermodynamic driving forces, making the ReaxFF-Hugoniostat framework a robust tool for predicting the atomic-level aging of polystyrene.

**Figure 6:**
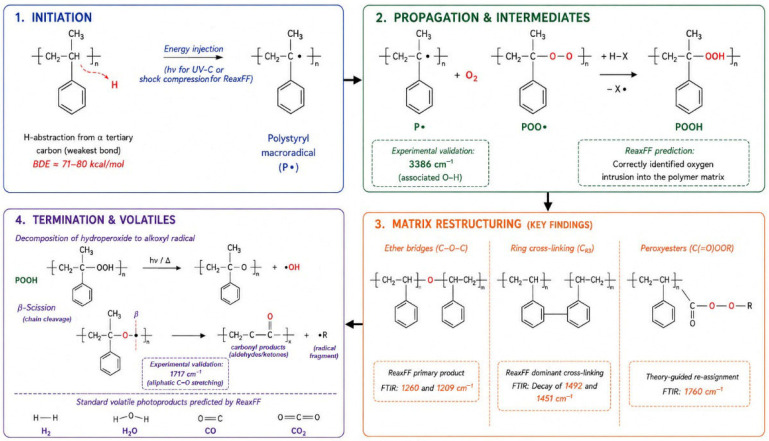
Proposed reaction mechanism for PS photo-oxidation in relation to ReaxFF predictions.

Figure 6 presents the identified degradation pathways in the form of a schematic diagram, facilitating the understanding of the reaction mechanisms and highlighting the observed synergy between experimental observations and ReaxFF simulations. The diagram is, of course, significantly simplified, particularly for the reactions occurring at higher compression pressures, where the processes proceed in a cascade-like manner, and the results are additionally influenced by stochastic factors, such as the random proximity of various molecular fragments.

## 4. Materials and Methods

### 4.1. Photodegradation of EPS Samples Under UV and Natural Sunlight

In order to investigate the degradation process of polystyrene, a series of expanded polystyrene (EPS) samples was prepared according to the following procedure. Packaging-grade EPS, free of any additives such as flame retardants, stabilizers (UV and thermal), or graphite/carbon black, was cut into strips with dimensions of 25 × 7 × 2 cm (L × W × T). Prior to irradiation, the samples were stored in light-tight containers.

**Figure 7:**
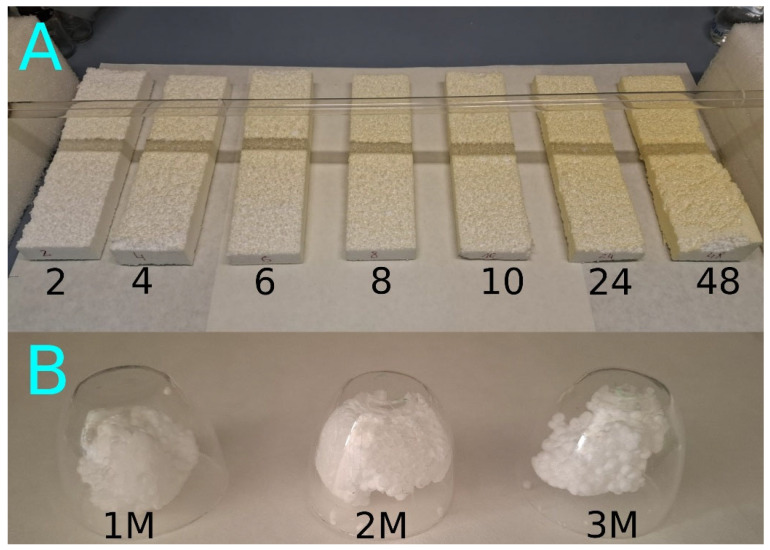
(**A**) EPS samples after exposure to UV-C radiation and (**B**) natural sunlight.

UV radiation was provided by a UV-C lamp (Philips TUV 36T5 HE 4P SE UNP/32, Signify, Eindhoven, Netherlands) emitting radiation in the UV-C range with a nominal power of 75 W. The EPS samples were arranged side by side at a distance of approximately 10 cm below the lamp (Figure 7). The samples were irradiated continuously for 2, 4, 6, 8, 10, 24, and 48 h.

After the specified irradiation time, each sample was transferred to a light-tight container. Subsequently, a fragment was collected from the area located directly beneath the UV lamp (for each sample, the fragment was taken from the same relative position) and subjected to FT-IR/ATR analysis. The polystyrene samples were designated as PS 2, PS 4, PS 6, PS 8, PS 10, PS 24, and PS 48. In addition, a non-irradiated reference sample was used as a control and labeled PS 0.

To compare accelerated degradation induced by UV-C irradiation with that caused by natural weathering, complementary experiments were conducted by exposing EPS to solar radiation. For this purpose, three fragmented EPS samples were placed in sealed quartz vessels transparent to UV radiation. The vessels were then positioned outdoors in a location characterized by high solar exposure, southern orientation, and a minimum daily irradiation time of 8 h. The exposure experiments were carried out for periods of 1, 2, and 3 months. After each exposure period, the samples were transferred to light-tight containers and subsequently analyzed by FT-IR/ATR spectroscopy. These samples were designated as PS 1M, PS 2M, and PS 3M.

### 4.2. FT-IR/ATR Analysis Methodology and Setup

FT-IR/ATR spectra were recorded using a Nicolet 6700 spectrophotometer (Thermo Fisher Scientific Inc., Madison, WI, USA) and a Harrick Meridian Diamond (Harrick Scientific Products, Inc., Pleasantville, NY, USA) attenuated total reflection attachment. The spectra were recorded in the range of 4000–400 cm^−1^, at a resolution of 4 cm^−1^. Each spectrum was the result of 256 co-added scans to achieve an optimal signal-to-noise ratio. The background was recorded using the clean, dry diamond crystal in contact with air prior to each sample application. To ensure the statistical reliability and reproducibility of the data, measurements for each sample were conducted at least in triplicate. All data acquisition and processing were managed using OMNIC^TM^ software (version 8.2.387, Thermo Fisher Scientific Inc., Madison, WI, USA). No smoothing functions were used.

### 4.3. Molecular Dynamics Simulation Protocol

The simulations were carried out using the LAMMPS molecular dynamics engine [44,49] compiled with the ReaxFF module [50], which is capable of handling ReaxFF force field parameter files. The molecular structure of polystyrene was initially prepared using the classical AMBER force field [41]. After structural folding, the system was transferred to a new simulation box, where degradation reactions were simulated using the ReaxFF force field potential with parameterization optimized for C/H/O/N-based polymers [26]. The simulation box was additionally filled with 100 oxygen molecules, which were initially placed at random positions. Partial charges on individual atoms were obtained by minimizing the electrostatic energy of the system using the charge equilibration (QEq) method, as described by Rappé and Goddard [51] and Aktulga et al. [50]. The integration timestep in all ReaxFF-based simulations was set to 0.25 fs. The degradation reactions were induced using Hugoniostat dynamics, as implemented in the *fix nphug* algorithm. Four shock compression pressures, 50, 60, 70, and 80 GPa, were applied, and the degradation reactions were simulated for 100 ps. Bond topologies were recorded every 2.5 ps. Subsequently, the systems were subjected to conventional molecular dynamics in the NVT ensemble at a temperature of 800 K for 250 ps in order to remove weak, transient bonds that typically form under such conditions. After subsequent cooling to 300 K, the resulting bond topologies were considered stable and ready for further analysis. The analysis of the simulation results was carried out using self-designed procedures that performed clustering of the system components based on information obtained from the output bond topologies, analysis of the chemical environment of each element, and comparison of the bond topologies with the initial structures.

## 5. Conclusions

The integration of experimental UV-C-accelerated aging with Reactive Molecular Dynamics (ReaxFF) simulations provides a robust and comprehensive framework for elucidating the atomic-level oxidative degradation mechanism of polystyrene. Although the physical modes of energy delivery differ—UV-C radiation via electronic excitation and shock compression via Hugoniostat dynamics—both processes are thermodynamically unified by the injection of energy required to overcome high activation barriers, such as the ~330 kJ mol^−1^ threshold for tertiary C–H bond scission. Our findings confirm that the ReaxFF-Hugoniostat approach acts as a valid numerical accelerator, allowing for the observation of primary radical-mediated restructuring within picosecond trajectories that correlate with experimental timescales. The synergy between theoretical predictions and experimental ATR-FTIR data is validated by several key chemical fingerprints:

1. The simulation identified extensive intermolecular ring-ring coupling (CR3) as a dominant mechanism. This was empirically confirmed by the systematic depletion of native aromatic skeletal bands at 1492 and 1451 cm^−1^ in the 48 h UV-aged samples, proving that ring fusion is a valid aging mechanism. However, we also note that the large number of CR3 cross-links may be partially facilitated by the high pressure experienced by the sample during shock wave propagation.
2. The ReaxFF model correctly predicted the formation of ether-type oxygen bridges (validated by bands at 1260 and 1209 cm^−1^) and peroxide intermediates. Specifically, the re-assignment of the 1760 cm^−1^ band to peroxyesters, supported by theory and recent literature, provides a more nuanced understanding of oxygen incorporation than traditional assignments.
3. While simulations identified high-abundance aldehyde precursors, the experimental observation of carboxylic acid dimers (3231 cm^−1^) highlights the complementary nature of this methodology—ReaxFF captures the primary oxidative steps, while FTIR confirms the secondary chemical endpoints that evolve over longer experimental periods.
4. The identification of H_2_, H_2_O, and CO in the simulation trajectories is consistent with established literature identifying these as standard volatile photoproducts of low-temperature PS aging, resulting from hydroperoxide decomposition and Norrish-type reactions, rather than being exclusive indicators of pyrolysis.

From an environmental perspective, the formation of these hydrophilic oxygen-containing groups is critical. The resulting increase in hydrophilicity enhances the bioavailability and potential biohazard of fragmented PS microplastics, underscoring the importance of understanding these complex atomic-level transformations for ecological risk assessment.

## Figures and Tables

**Table 1:** Essential parameters of the PS nanoparticle before and after degradation induced by shock compression at various pressures. New chemical connections are labeled according to standard chemical nomenclature and are presented as structural formulas in Figure 5. Specifically: OH—hydroxyl groups; C–O–C—ether linkages; CHO—aldehyde groups; C=O—carbonyl groups; R3, R4, R5, R6—heterocyclic rings containing oxygen, with the number indicating ring size; CR3—cross-links formed between carbon atoms from benzene rings; CR4—cross-links formed when a carbon atom with 3 bonds to other carbons gains a fourth bond after degradation with another carbon atom; C–OO—terminal peroxy groups; C–OO–C—peroxy linkages. Rg is the radius of gyration of the nanoparticle, SASA is the solvent-accessible surface area, and ALT denotes atoms whose chemical environment has been altered in any way due to shock compression at the given pressure.

		Compression Pressure			
		50 GPa	60 GPa	70 GPa	80 GPa
New chemical connections/ functional groups within/on the nanoparticle	ALT	0	53	490	2509
	OH	0	1	4	29
	C–O–C	0	0	16	53
	CHO	0	2	9	48
	C=O	0	1	1	9
	R3	0	0	2	4
	R4	0	0	1	0
	R5	0	0	2	5
	R6	0	0	1	5
	CR3	0	39	334	1178
	CR4	0	1	26	121
	C–OO	0	0	3	3
	C–OO–C	0	1	3	1
Big species		3xC_1546_H_1550_	C_1546_H_1550_O_4_ C_3092_H_3100_O_2_	C_4636_H_4648_O_44_	C_4527_H_4470_O_160_
Small species		-	-	C_2_H_2_O_2_ (glyoxal)	C_22_H_20_O; C_13_H_12_O_2_; C_8_H_8_O_2_; C_8_H_8_; C_7_H_5_O; C_7_H_6_;C_6_H_5_
Rg, nm		2.192	2.114	2.282	2.032
SASA, nm^2^		176.5	168.4	196.2	177.6

**Table 2:** Correlation between theoretical predictions and experimental FT-IR bands.

Predicted Group (Theory)	Experimental FT-IR Peak (cm^−1^)	Spectral Assignment
Ring-ring connections (CR3)	1492, 1451 (decay)	Polymer matrix cross-linking
Ether bridges (C–O–C)	1260, 1209	C–O stretching in esters/skeletal
Aldehydes/ketones	1717	Aliphatic C=O stretching
Peroxyesters/peroxides	1760	C=O stretching in peroxyesters
Hydroxyls (OH)	3386, 3231	O–H stretching/acid dimers
Peroxides (O–O)	3386 (broad)	Hydroperoxide (PS–OOH)

## Data Availability

Data are available upon request.
